# PP2A and Its Inhibitors in Helper T-Cell Differentiation and Autoimmunity

**DOI:** 10.3389/fimmu.2021.786857

**Published:** 2022-01-05

**Authors:** Mohd Moin Khan, Ubaid Ullah Kalim, Meraj H. Khan, Riitta Lahesmaa

**Affiliations:** ^1^ Turku Bioscience Centre, University of Turku and Åbo Akademi University, Turku, Finland; ^2^ InFLAMES Research Flagship Center, University of Turku, Turku, Finland; ^3^ Turku Doctoral Programme of Molecular Medicine (TuDMM), University of Turku, Turku, Finland

**Keywords:** PP2A, inflammatory, PP2A activating drugs, T cell differentiation, autoimmune disease

## Abstract

Protein phosphatase 2A (PP2A) is a highly complex heterotrimeric Ser/Thr phosphatase that regulates many cellular processes. The role of PP2A as a tumor suppressor has been extensively studied and reviewed. However, emerging evidence suggests PP2A constrains inflammatory responses and is important in autoimmune and neuroinflammatory diseases. Here, we reviewed the existing literature on the role of PP2A in T-cell differentiation and autoimmunity. We have also discussed the modulation of PP2A activity by endogenous inhibitors and its small-molecule activators as potential therapeutic approaches against autoimmunity.

## Protein Phosphatase 2A (PP2A)

Protein phosphorylation is a post-translational modification (PTM) and is indispensable in cell signaling regulation. Mechanistically, by altering the charge on a protein, phosphorylation alters the conformation, which alters the protein’s subcellular localization, interactions with other proteins, and functions. Protein phosphorylation is regulated by enzyme kinases and phosphatases, which catalyze phosphate’s addition or removal, respectively. The altered activity of these enzymes is one of the major defects in the development of various diseases, such as cancers, neurological and autoimmune disorders (1–3).

Although kinases and phosphatases regulate protein phosphorylation, the focus has been on kinases for several reasons: more genes encode kinases, and the consideration that phosphorylation acts as a response to perturbation while dephosphorylation as mean to restore equilibrium (4). However, recent studies indicate that phosphatase inhibition is also a common signature in several human diseases (4). In addition, the phosphatases are inherently more complex due to the combinatorial diversity of phosphatase regulatory subunits that results in a greater functional number of phosphatases. Nevertheless, there is a growing realization that phosphatases are equally important and hold therapeutic potential for disease treatments.

In this review, we will discuss the role of protein phosphatase 2A (PP2A) in T-cell differentiation and autoimmunity. The tumor-suppressive function of PP2A has been reviewed elsewhere (4–10). PP2A is a highly conserved serine/threonine heterotrimeric phosphatase with an essential role in many cellular processes (4). PP2A activity is inhibited in several cancers (8, 9, 11). Multiple mechanisms have been proposed for the altered PP2A activity in cell transformation in cancer (9, 10, 12). In addition, PP2A is inhibited in neuroinflammatory and neurodegenerative diseases, such as Alzheimer’s disease and Parkinson’s disease (13–19).

**Figure 1:**
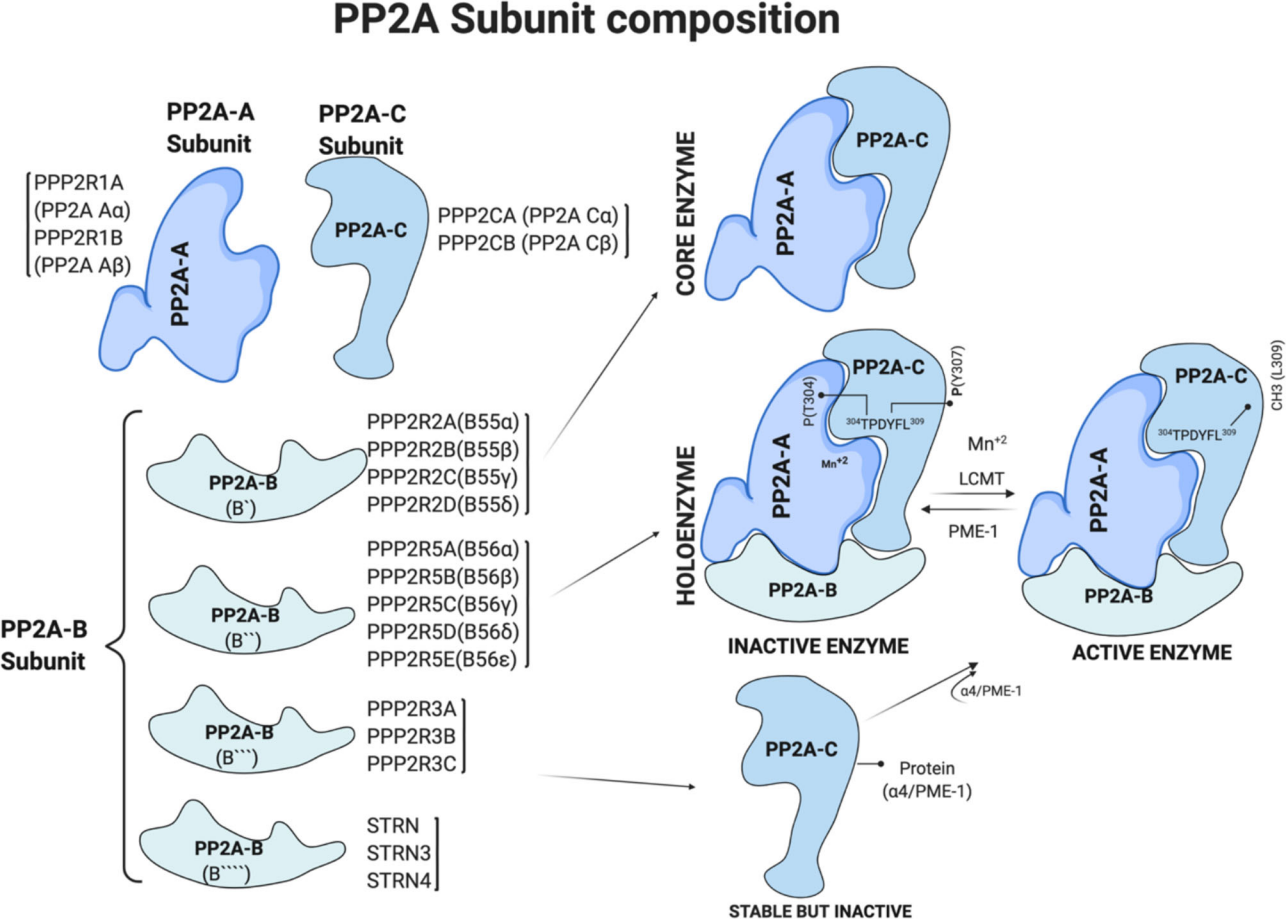
PP2A subunit composition and regulation of PP2A holoenzyme by post translational modification. Generated based on (4, 7). **Figure 1** is modified from the thesis (24) and prepared using BioRender.com.

PP2A contains three subunits. The “A” scaffolding subunit and “C” catalytic subunit together form a dimer of the core enzyme. A variable size “B” regulatory subunit binds the AC dimer. These subunits exist either as AC dimers, ABC trimers (called holoenzyme), or free inactive catalytic C subunits stabilized due to interactions with protein PME-1 or α4, also known as immunoglobulin binding protein 1 (IGBP1) (20–23) (**Figure 1**). In humans, the A, B, and C PP2A subunits are located on different chromosomes, and isoforms of each subunit, especially B subunits, form the diversity of the PP2A enzymes.

Expression of the A and C subunits is ubiquitous and promiscuous. Two distinct and non-redundant genes *PPP2R1A* and *PPP2R1B*, encode A subunits, PP2A Aα and PP2A Aβ, respectively. Similarly, *PPP2CA* and *PPP2CB* encode catalytic C subunits, PP2A Cα and PP2A Cβ, respectively (**Figure 1**). Higher levels of expression of α isoforms than the β isoforms of A and C subunits in most human tissues is due to a 7–10-fold stronger promoter of their respective genes (25, 26). The PP2A-A subunit has the characteristic 15 HEAT (Huntingtin, Elongation factor, the A subunit of PP2A, and Target of Rapamycin) repeats of antiparallel alpha-helices (5). These repeats stack to form an extended loop-like structure with a highly flexible hinge region between helices 12 and 13 that creates groves for the catalytic C and regulatory B subunit binding (5, 27–29).

The B subunits confer enzyme-substrate specificity, and their expression and localization vary with the cell type, creating diversity. PP2A-B subunits are classified into four groups: **B** [*PPP2R2A*, *PPP2R2B*, *PPP2R2C*, and *PPP2R2D*), **B′** (*PPP2R5A*, *PPP2R5B*, *PPP2R5C*, *PPP2R5D*, and *PPP2R5E*), **B″** (*PPP2R3A*, *PPP2R3B*, and *PPP2R3C*), and **B″′** (*STRN*, striatin family)]. The B subunits proteins are structurally different in each group, and their binding to the PP2A holoenzyme is due to the intrinsic flexibility of the scaffolding A subunit. Further, the binding of the B subunit to the dimer is mutually exclusive, i.e., only one B subunit at a time interacts with the dimer to form a holoenzyme (30). **Figure 1** summarizes the subunits and genes that encode them.

## Regulation of PP2A Activity

PP2A activity is regulated at the level of gene expression, generation of splice variants, and combinatorial diversity due to a large number of regulatory subunits. Notably, the generation of PP2A active holoenzyme in cells is tightly regulated to avoid the formation of enzymes with impaired substrate specificity. Besides these, PP2A activity is also regulated by post-translational modification (PTM) of different subunits or interaction with other proteins. The carboxy-terminal of the PP2A catalytic C subunit is the hotspot for PTM (31).

Methylation on leucine-309 (L309) residue of C subunit is well known to control PP2A enzymatic activity and composition (29, 31) (**Figure 1**). Leucine carboxyl methyl transferase 1 (LCMT1) uses S-adenosylmethionine (SAM) as a substrate to add methyl group at leucine-309 (L309). On the other hand, protein methyl-esterase 1 (PME-1) removes the methyl group from the L309 residue, thereby inhibiting the catalytic activity of PP2A (31) (**Figure 1**). Crystal structure studies of the PP2A-PME complex showed a non-methylated L309 residue at a negatively charged carboxyl group prevents heterotrimer formation and thus determined the composition of the PP2A holoenzyme. Methylation of L309 residue of the catalytic subunit is essential for PPP2R2A (B55α) binding with PP2A-A-C dimer to form the holoenzyme. In addition to methylation, phosphorylation on tyrosine-307 (Y307) and threonine-304 (T304) residues of the C subunit negatively regulate PP2A enzymatic activity. However, antibodies detecting phosphorylated Y307 are non-specific, and a fuller understanding will require antibody-independent mass-spectroscopy-based methods.

PTM of PP2A regulatory B subunits has also been reported. For instance, phosphorylation of the PPP2R5D subunit by protein kinase A led to an increase in PP2A activity (4, 32). Extensive phosphorylation of PPP2R5 and STRN family members was reported in unbiased proteomics studies (33). Besides, C terminal lysine residues of both PPP2R2A and PPP2R2D are also acetylated (4, 34, 35). However, further studies are required to confirm the role of phosphorylation and acetylation of regulatory subunits on PP2A activity.

PP2A activity is also regulated by its interactions with other proteins. PP2A-C is activated by binding of phosphotyrosyl phosphatase activator (PTPA) (36–38). PTPA releases PP2A-C from PME-1-mediated inhibition (22). Further, PTPA binding to the A-C dimer facilitates conformational changes required for holoenzyme formation (36–38). PME-1-mediated regulation of PP2A activity has been reviewed elsewhere (31).

## PP2A Inhibitors

PP2A activity is regulated by endogenous inhibitors encoded in the genome as well as chemical small molecule inhibitors. PP2A endogenous inhibitors acidic nuclear phosphoprotein 32A (ANP32A), also known as PP2A inhibitor 1, and SET, also known as PP2A inhibitor 2 are members of the SET family. SET directly interacts with PP2A-C to suppress its activity, and enhanced SET expression is associated with several cancers (39).

Cancerous inhibitor of protein phosphatase 2A (CIP2A; KIAA1524; p90) is another endogenous inhibitor of PP2A. CIP2A-PP2A interaction prevents c-Myc S62 dephosphorylation and proteolytic degradation, leading to cell transformation (7, 40). Structural studies showed CIP2A N terminus interacts with PP2A-B56 subunits PPP2R5A (B56α) and PPP2R5C (B56γ) (41). CIP2A dimerization contributes to maximal binding to the B56 subunit. This binding inhibits holoenzyme formation (41). CIP2A also appears to bind to the A and C subunits (42), whose functions remain to be studied. PP2A inhibition by CIP2A overexpression in Alzheimer’s disease (43) leads to the accumulation of hyperphosphorylated tau protein aggregates and Alzheimer’s development. Another study identified a non-coding RNA LINC00665 encoded micro peptide called CIP2A-BP (CIP2A binding peptide), which interacts with CIP2A N-terminus and compete for B56γ binding site to release PP2A (44).

Proteins PME-1 and α4 are also considered as PP2A inhibitors by controlling heterotrimeric holoenzyme assembly. PME-1 overexpression is reported in cancer cells, whereas its inhibition enhanced PP2A activity (31).

Okadaic acid is a naturally occurring small-molecule inhibitor of phosphatases, including PP2A. Okadaic acid is isolated from the marine dinoflagellates and causes shellfish poisoning (45). Although OA inhibits other serine-threonine phosphatases, its specificity for PP2A is high at low concentrations (46). LB100 is another small-molecule inhibitor of PP2A. However its specificity for PP2A has been questioned (47).

## PP2A Activators

Researchers have tried various compounds to activate PP2A as potential cancer treatments. Some activate PP2A indirectly by inhibiting its inhibitors. For instance, small-molecule PME-1 inhibitors aza-β-lactam (ABL) and sulfonyl acrylonitrile are associated with PP2A activation (48, 49). Similarly, inhibitors of CIP2A, such as Celastrol (tryptamine), anti-cancer drug bortezomib, and dipeptidyl boronic acid induce PP2A activity towards its targets. Forskolin, carnosic acid, and vitamin E analogs, such as α-tocopherol succinate, also activate PP2A through uncharacterized mechanisms (5).

Cell-endogenous lipid metabolite ceramide activates PP2A in immune and cancer cells. Ceramides de-repress PP2A activity by direct interaction with PP2A endogenous inhibitor SET (50, 51). The common immunosuppressive lipid sphingosine analog drug Fingolimod (2-amino-2-[2-(4-octylphenyl) ethyl] propane-1,3-diol), also known as FTY720, which is derived from myriocin (ISP-1), a metabolite of fungus *Isaria sinclairii*, also activates PP2A (52–57). In mice, FTY720-mediated activation of PP2A was identified as a promising strategy in reducing tumor and cell transformation.

Anti-psychotic phenothiazine drugs, such as chlorpromazine also activate PP2A. However, their anti-cholinergic effects limit their use as anti-cancer agents. Further developments in this line led to identification of small-molecule activators of PP2A (SMAPs), such as DBK-1154, DT-061, and iHAPs (improved heterocyclic activators of PP2A). SMAP binding stabilizes and promotes PP2A heterotrimeric holoenzyme assembly with robust activation (4, 58–60). DT-061 overcomes PP2A endogenous inhibition in cancer cells by binding to small pockets and act as a “glue” to keep PP2A subunits together for heterotrimeric functional PP2A enzyme (61).

## PP2A in CD4+ T-Cell Activation and Differentiation

PP2A is required to limit T-cell activation. It limits protein kinase C (PKC-θ) dependent CARMA1 phosphorylation, which recruits signaling mediators important for T-cell activation (62). Both in resting and activated T cells, PP2A regulatory subunit Aa (PPP2R1A) interacts with CARMA1 leading to dephosphorylation. Consistent with this idea, PP2A inactivation in Jurkat T cells and murine Th1 cells results in enhanced Carma1 S645 phosphorylation and NF-κB activation and IL-2 and IFN-γ production. In addition, depletion of endogenous PP2A inhibitor, CIP2A, led to reduced T-cell activation (63). NF-κB is a critical transcription factor regulating the expression of immune response genes, but its aberrant activity contributes to autoimmunity (64). PP2A regulatory subunit B56γ is strongly upregulated upon T cell activation and acts as a negative regulator of NF-κB by dephosphorylating IKK in TCR signaling. B56γ silencing in T cells, increased NF-κB activity and enhanced expression of inflammatory genes and T cell proliferation (64).

PP2A also contributes to CD4+ T-cell differentiation. Genetic deletion of a PP2A regulatory subunit PPP2R2A in mice led to reduction in Th1 differentiation *in vitro* as measured by IFNγ expression (65). Interestingly, PP2A inhibition by a small molecule PP2A inhibitor LB100 led to increase in Th1 differentiation in mice (66). The contradictory results obtained upon genetic deletion of PPP2R2A and competitive PP2A inhibition by LB100 could possibly be due to lack of specificity of the compound (47) or due to differential activity of regulatory subunits. Effect of PP2A on Th2 differentiation has been less studied, however the treatment with LB100 led to reduction in IL-4 expression and increase in IFNγ by Th2 cells (66). PP2A inhibition by okadaic acid or LB100 led to reduction in IL-9 production by Th9 cells (67).

Depleting PP2A catalytic subunit α in mice led to a reduction in Th17 cell differentiation and development of experimental autoimmune encephalomyelitis (EAE) (68). PPP2A regulates SMAD2/3 phosphorylation and RORγt binding on *IL17A* in Th17 cells. Glomerulonephritis developed in transgenic animals with PP2A catalytic subunit (PP2Ac) overexpression in an IL-17-dependent manner (69). Mechanistically, enhanced IL-17 is due to the PP2Ac-mediated activation of Rho kinase (ROCK), which phosphorylates transcription factor IRF4 and leads to binding and recruitment of histone acetyltransferases (HAT) and other factors at the *IL17* locus for enhanced histone 3 acetylation and increases gene expression (69, 70).

In contrast to mice, silencing the PP2A scaffolding subunit A in human Th17 cells or inhibiting its activity by okadaic acid upregulated IL17 expression (71). Additionally, PP2A activation by FTY720 led to a reduction in IL17A expression in human Th17 cells. Similar effects of FTY720 treatment were seen in sera from patients with multiple sclerosis (MS) (72). Therefore, targeting PP2A in mice and human Th17 cells appears to have the opposite effect on IL17A expression. This difference is perhaps due to species-specific differences, as has been reported earlier for chromatin modifier SATB1 (73). Also, a small overlap in Th17 signature genes between human and mouse Th17 cells suggests significant differences in the Th17 cell transcriptome of the two species (74). Interestingly, both in human and mouse Th17 cells, CIP2A negatively regulates IL17A expression and STAT3 phosphorylation (71). In human Th17 cells, depletion of both PP2A and CIP2A results in enhanced IL17A expression, suggesting that CIP2A may regulate IL17A expression in a PP2A independent manner.

**Figure 2:**
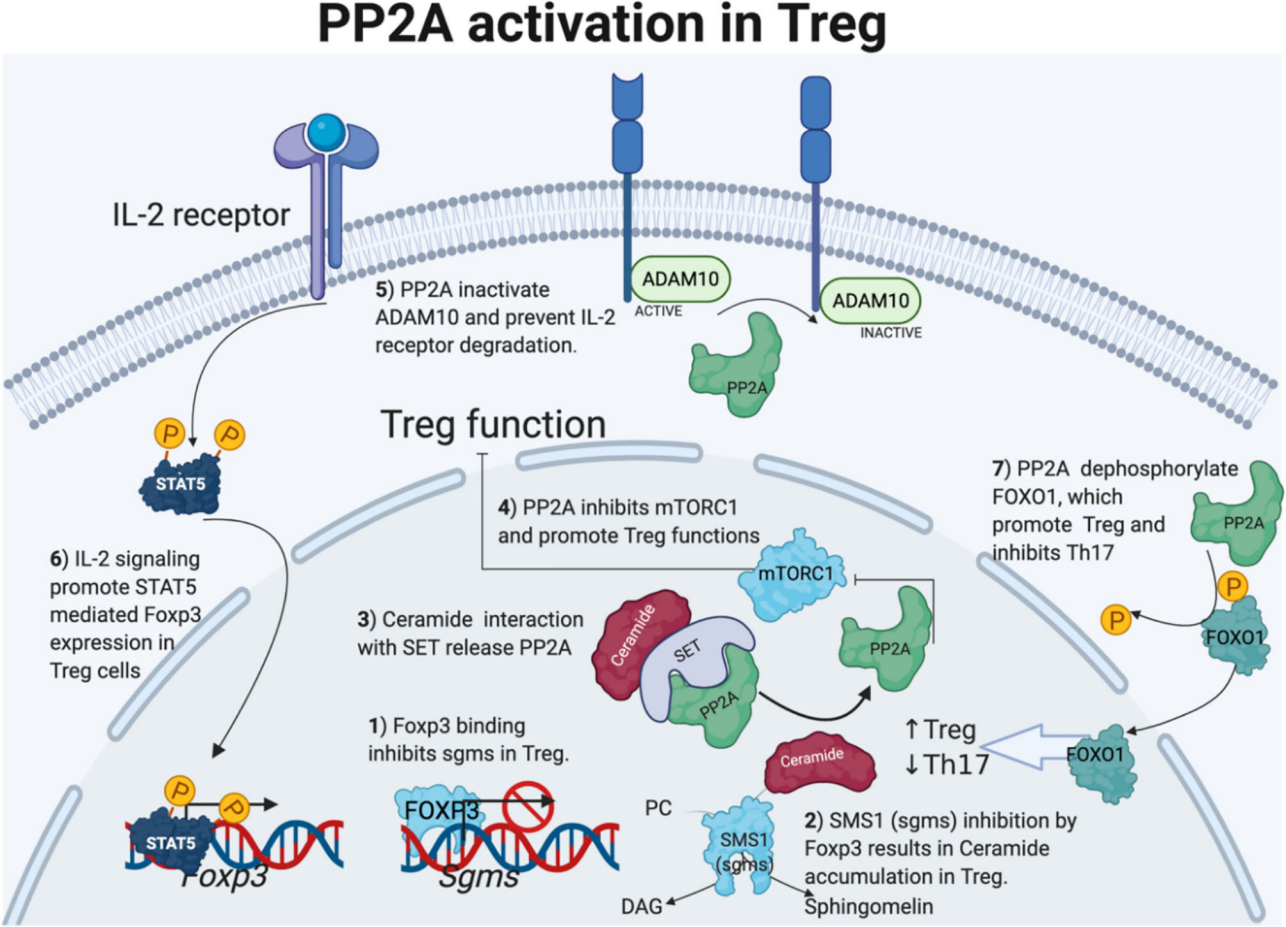
PP2A activation in Treg cells. Foxp3 direct sgms1 gene promoter binding and inhibits sgms1 expression in Treg. SMS1 (encoded by sgms1) reduction results in ceramide accumulation. Ceramide accumulates only in Treg cells, and the interaction with TCR-activated PP2A endogenous inhibitor SET activates PP2A. On the other hand, PP2A activity in Treg cells is important to inhibit mTORC1 and ADAM10 to the IL-2 receptor. Enhanced IL-2 signaling promotes STAT5 and Foxp3 expression in Treg cells. Lastly, PP2A dephosphorylation activates Foxo1, which positively regulates Treg-cell differentiation but inhibits Th17 cells. **Figure 2** is modified from the thesis (24) and prepared using BioRender.com.

PP2A activity is also required for the Treg cell-mediated suppression of effector T cells responses (75, 76). Treg-specific deletion of PP2A results in multiorgan autoimmunity in animals (75). Mechanistically, Foxp3 binds to *Sgms1* promoter that encodes phosphatidylcholine:ceramide choline phosphotransferase 1 (SMS1) and inhibits its expression. Reduced SMS1 expression leads to the accumulation of ceramide in Treg cells as SMS1 takes choline and ceramide as substrates to make DAG and sphingomyelin. Ceramide binds to SET and releases PP2A from SET-mediated inhibition, leading to enhanced PP2A activity in Treg cells. Enhanced PP2A activity inhibits mTORC1 and promotes Treg suppression of effector T cells. Enhanced PP2A activity also inhibits activity of sheddase ADAM10, which cleaves the IL2 receptor (69, 76). Therefore, higher PP2A activity prevents IL-2R degradation and promotes STAT5 phosphorylation and Foxp3 expression in Treg (69). **Figure 2** summarizes the ways through which PP2A activation supports Treg cell function.

PP2A-mediated Treg modulation through Foxo1, another transcription factor is also fascinating. FOXO1 inhibits transcriptional activity of RORγt to its target genes, such as Il17a and Il23r (77) and inhibits EAE development (78). Interestingly, FOXO1 binds to Foxp3 promoter and its conserved non-coding sequence 2 element and promotes Foxp3 expression in T cells. PP2A-mediated Foxo1 dephosphorylation results in translocation of Foxo1 to the nucleus and transcriptional activation (**Figure 2**). PP2A-mediated Foxo1 activation and modulation of Treg-cell function is an exciting area for further research Interestingly, Foxo1 is highly expressed in lymphoid cells and controls T-cell homing to secondary lymphoid organs. Foxo1 deficiency causes diminished expression of trafficking molecules, including S1PR1 (79).

## PP2A in Autoimmunity

PP2A has not been extensively studied in the context of autoimmunity. However, owing to its important role in T cell activation and differentiation PP2A may be important for development of autoimmunity. MS is an autoimmune disease characterized by immune-mediated destruction of the myelin sheath of neurons. Myelin-reactive T cells egress from the lymph node and migrate to central nervous system to Sphingosine-1-phosphate (S1P) gradient, which is high in body fluids and tissues, including the central nervous system. Current MS treatment seeks to minimize lymphocyte egress and migration across the blood-brain barrier (BBB) to prevent inflammation and destruction of neurons. FTY720, an analog of S1P, binds to S1PR on lymphocytes and is a common immunosuppressive compound that is approved to treat MS. It has been proposed to act through S1PR internalization and degradation in autoreactive lymphocytes, rendering the lymphocytes unresponsive to the S1P gradient.

Besides regulating the egress and migration of autoreactive T cells to the central nervous system, FTY720 may also limit MS by activating PP2A in T cells. However, whether PP2A activation and immunosuppressive function are inter-related remains to be clarified. Phosphorylated FTY720 is considered immunosuppressive and different from the non-phosphorylated PP2A activators and antitumor forms (80–82). The hypothesis is mainly based on the fact that non-phosphorylated FTY720-derivative, 2-amino-4-(4-heptyloyphenol)-2-methylbutanol [AAL(S)] treatments failed to reduce EAE disease in animals (83, 84). Further, AALs, MP07-66, OSU-2S, P053 have limited S1PR binding (81, 85–88). Enrichment of proinflammatory Th17 cells has been noted in MS lesions, and its inflammatory cytokine, IL-17, was implicated for MS pathogenesis (89). Th17 disrupts the BBB and promotes CNS inflammation (90). Interestingly, FTY720 treatment reduced Th17 levels in peripheral blood of MS patients (91, 92). In addition, Fingolimod treatment decreases peripheral blood proinflammatory Th1/17 cytokines levels in patients with multiple sclerosis, whereas it increased the frequency of Treg cells (72). Further, in human Th17 cells, we found that PP2A-A subunit silencing or okadaic acid treatments upregulate IL17 expression, but FTY720 treatments reduce IL-17 levels (71). Therefore, the possibility of PP2A activation in FTY720-immunosuppressive functions cannot be ruled out. Also modification of the original FTY720 compound, such as blocking phosphorylation site, may completely change the properties of the compound, and it may not bind to S1PR or treat EAE.

Besides MS, PP2A also has a role in other autoimmune diseases. Systemic lupus erythematosus (SLE) is an autoimmune disease characterized by widespread inflammation due to immune cell activation. Like MS, SLE is also ameliorated by FTY720 treatment in a mouse model (93). PP2A activator, AAL, treatment prevents disease in an animal model of rheumatoid arthritis (94). In line with this, the CIP2A inhibitor celastrol reduces rheumatoid arthritis in animals and is anti-inflammatory (95, 96). These findings suggest a potential for PP2A modulation in treating autoimmune disease. The use of a recently developed small-molecule activator of PP2A for the treatment for other autoimmune diseases should be tested.

## Conclusions

PP2A is one of the most abundant proteins in the cell and the most prominent serine-threonine phosphatase. It controls a range of cellular processes and is highly conserved from yeast to mammals and is required for T-cell activation. PP2A has a complex role in CD4+ T cell differentiation to distinct subsets. However, it clearly promotes Treg differentiation, suggesting PP2A activation as an attractive strategy for immunosuppression. The recent development of small-molecule compounds that directly activate PP2A might provide means for the development of therapeutics for immune cell-mediated diseases. However, further work is needed to study specificity and mode of action of these compounds.

## Author Contributions

All authors listed have made a substantial, direct, and intellectual contribution to the work and approved it for publication.

## Funding

MMK was supported by the University of Turku graduate school on Turku Doctoral Programme of Molecular Medicine (TuDMM) and a central grant from the Finnish Cultural Foundation. UK was supported by Varsinais-Suomi regional Fund from Finnish Cultural Foundation. RL was supported by the Academy of Finland, AoF, Centre of Excellence in Molecular Systems Immunology and Physiology Research (2012-2017) grant 250114; by the AoF grants 292335, 294337, 292482, 31444, 335435, 331793, 329277, and by grants from the JDRF, the Sigrid Jusélius Foundation, the Jane and Aatos Erkko Foundation and the Finnish Cancer Foundation.

## Conflict of Interest

The authors declare that the research was conducted in the absence of any commercial or financial relationships that could be construed as a potential conflict of interest.

## Publisher’s Note

All claims expressed in this article are solely those of the authors and do not necessarily represent those of their affiliated organizations, or those of the publisher, the editors and the reviewers. Any product that may be evaluated in this article, or claim that may be made by its manufacturer, is not guaranteed or endorsed by the publisher.
